# Nano-TiO_2_ Doped Chitosan Scaffold for the Bone Tissue Engineering Applications

**DOI:** 10.1155/2018/6576157

**Published:** 2018-09-03

**Authors:** Pawan Kumar

**Affiliations:** Department of Materials Science and Nanotechnology, Deenbandhu Chhotu Ram University of Science and Technology, Murthal-131039, India

## Abstract

The present focus is on the synthesis of highly effective, porous, biocompatible, and inert scaffold by using ceramic nanoparticles and natural polymer for the application in tissue engineering. Freeze-drying method was used to fabricate nano-TiO_2_ doped chitosan sample scaffold. Nano-TiO_2_/chitosan scaffold can considered as an effective solution for damaged tissue regeneration. The interaction between chitosan (polysaccharide) and nano-TiO_2_ makes it highly porous and brittle that could be an effective substitute for bone tissue engineering. The TiO_2_ nanoparticles have a great surface area and inert properties while chitosan is highly biocompatible and antibacterial. The physiochemical properties of TiO_2_ nanoparticles and scaffold are evaluated by XRD and FTIR. The nanoparticles doped scaffold has given improved density (1.2870g/cm^3^) that is comparatively relevant to the dry bone (0.8 - 1.2 gm/cm^3^). The open and closed porosity of sample scaffold were measured by using Brunauer–Emmett–Teller analyzer (BET) and scanning electron microscopy (SEM). The mechanical properties are examined by stable microsystem (Texture Analyzer). The in vitro degradation of scaffold is calculated in PBS containing lysozyme at pH 7.4. Electron and fluorescence microscopy are used to study morphological characteristics of the scaffolds and TiO_2_ nanoparticles. The growth factor and drug-loaded composites can improve osteogenesis and vascularization.

## 1. Introduction

In the human body, bone is an extremely dynamic and diverse tissue (structurally and functionally). The human skeletal system is a collection of long to short, flat, and irregular bones. The average pore sizes of bone are 100-300 *μ*m. The active diffusion of nutrients occurs within 150-200 *μ*m pore sizes from blood supply. The nonmineralized (type-1 collagen) and mineralized phases (plate like apatite) are main composition of bone extracellular matrix [1–3]. Trauma, injury, infections, and bone extracellular matrix (ECM) loss are among the most human health threatening problems. Bone tissue engineering easily eliminates the issues of surgical treatments including donor site morbidity, inadequate availability, immune response of the body, and infections. The different substitutes including hydroxyapatite, different composition of bioactive glass, a number of synthetic and natural polymers, and their composites were fabricated for the bone tissue engineering. Still, none of the above materials could meet all the characteristics of bone graft substitutes [4, 5]. The three major approaches of tissue engineering include isolated bone cells (osteogenic) transplantation or injection to the desired injured site, application of isolated tissue inducing molecules, or growth factors to the defected site and designing of 3D scaffolds [6]. Chitosan (natural polysaccharide) is the derivative of chitin (most abundant in nature after cellulose), used as graft material in tissue restoration because of its high biocompatibility and rapid degradation without toxicity. The well-designed 3D scaffold having the capability to mimic the extracellular matrix of bone promotes cell adhesion and cell proliferation without producing toxicity and helps in the promotion of new tissue [7, 8]. The progress of any scaffold depends on its porosity and biomechanical and physical properties that allows fast vascularization without producing cytotoxicity. The chitosan scaffold exhibits cationic nature, which is liable for exchange of negatively, charged molecules like proteoglycans, glycosaminoglycan (GAG), and other nutrients [9]. GAG is the chief constituent of ECMs that allow cell accumulation or adhesion and proliferation on the surface of scaffold. Many studies stated that the porous structure [10] gels [11], thin films [12], membranes [13], and fibres [14] favour more bone cell growth with chitosan. However, chitosan has numerous shortcomings such as lack of mechanical strength, fast degradation, and missing of cell signaling molecules that are most important for growth of damaged tissue [15, 16]. Titanium oxide (TiO_2_) is available in the form of nanocrystals having a high surface area. Some researcher [17] observed the antiseptic and antibacterial activity of titanium oxide. Some researcher also proposed that TiO_2_ can be used as good filler materials in biodegradable polymers because the presence of titanium oxide enhances cell attachment and cell proliferation [18]. Due to its inert property, it has medical and health applications. Both chitosan and TiO_2_ nanoparticles are biocompatible, inert, and chemically stable so that they can be useful for hard tissue engineering also. They support cell adhesion and proliferation without producing toxicity. A lightweight and cost-effective TiO_2_/chitosan scaffold can be fabricated by freeze-drying method that fulfills many biomechanical requirements of a hard tissue graft. The present work includes TiO_2_ nanoparticles doped chitosan for scaffold synthesis.

## 2. Materials and Methods

### 2.1. Materials

Analytical grade low molecular weight chitosan (75-85% deacetylated), titanium tetra isopropoxide (TTIP, 97% assay), sodium borohydride, glutaraldehyde (25% in H_2_O), acetic acid, and NaOH were purchased Sigma-Aldrich. Lysozyme was procured from Thermo Fisher Scientific.

### 2.2. Synthesis of TiO_2_ Nanoparticles

The hydrothermal method is used for the TiO_2_ nanoparticles synthesis. Firstly, 1M titanium tetra isopropoxide (TTIP) is used to make colloidal solution by hydrolysis, mixed with acetic acid (4M), and allowed for one hour of stirring. After an aging period of 24 hours, 25 ml of this solution transferred to Teflon lined tube of stainless steel autoclave and placed in oven at 180°C for 12 hours. After that, precipitates were washed three times with distilled water. The mixture solution was filtered and placed in oven for drying at 100°C to get TiO_2_ crystal [19].

### 2.3. Scaffold Synthesis

**Figure 1:**
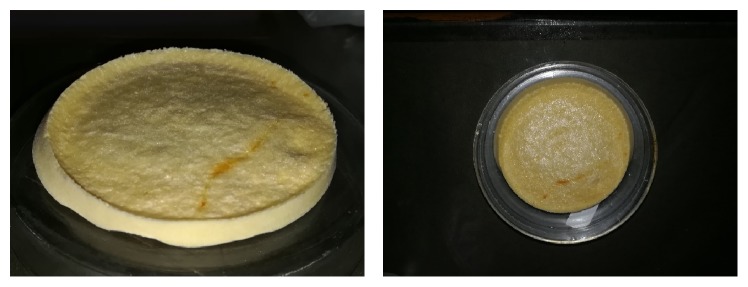
Lyophilized sample scaffold of chitosan/TiO_2_.

For the synthesis of chitosan/TiO_2_ sample scaffold, firstly make 2% (w/v) solution of chitosan by the addition of 1% (w/v) acetic acid solution with 6 h stirring. The calculated amount (1g) of titanium oxide nanoparticles is mixed with 100 ml distilled water to make slurry. Add this slurry in viscus solution of chitosan with continuous stirring for 12 h at room temperature. 1 M solution of NaOH is used dropwise to adjust pH 10 of the mixture. 0.5 ml solution of glutaraldehyde (0.25% v/v) is used to make cross-linking between chitosan and TiO_2_. After 15 hours of stirring, degas the suspension centrifugally and dispense it into Petri plates. These Petri plates are placed at - 80°C in an ultralow temperature freezer (LFZ-86L series, LABFREEZ) for 72 hours. After 72 hours, these frozen samples were transferred to the chamber of lyophilizer (ALLID FROST, Macflow Engg. Pvt. Ltd.), where the ice was removed by direct sublimation and the unfrozen water removed by desorption in a secondary drying process [20]. The freeze-dried sample neutralized with distilled water to remove acetate residue. The free glutaraldehyde (uncross-linked) was removed by using 5% sodium borohydride (reducing agent) solution and then washed with distilled water. After that, lyophilize these samples again for 48 h and collect pale yellow color scaffolds as shown in Figure 1.

### 2.4. XRD Analysis

The graphical X-ray powder diffraction patterns of the TiO_2_ and chitosan/TiO_2_ samples were noted down by Rigaku Ultima IV X-Ray diffractometer, using Cu-Ka (1.5406 Å) radiation at room temperature in the range of 10° to 60° at 2 theta degree scale.

### 2.5. FTIR Analysis

The Fourier transform infrared (FTIR) spectra (from 4000 cm^−1^ to 400 cm^−1^) of the hydrothermally synthesized TiO_2_ nanoparticles and chitosan/TiO_2_ scaffold were recorded in Perkin Elmer Spectrum RX1 spectrometer. FTIR gives the information related to the presence of different chemical or functional groups in the samples.

### 2.6. SEM Observation

Scanning electron microscope (SEM, JEOL) has given the information about the surface and the fracture section of scaffold. For the SEM analysis, thin section of sample scaffold was used with gold prior coating.

### 2.7. TEM Observation

Transmission Electron Microscope produces high-resolution images by the transmission of high-energy electron through the specimen. Structure, composition, and size of nanoparticles were analyzed by TEM (Hitachi H 7500) results. These nanoparticles were used for doping with chitosan to make scaffold.

### 2.8. Bulk Structure Analysis

Fluorescence Microscope (Leica DM 250) studied the surface topography and distribution of TiO_2_ nanoparticles in chitosan gel. The chitosan/TiO_2_ gel was used to understand the connectivity and branched structure existing between chitosan and TiO_2_.

### 2.9. Porosity Measurement

The pore volume, micropore radius, and pore specific surface area of the sample were examined by BET (Quantachrome® Nova Station). The small sized pieces of sample scaffold were loaded in the sample tube and set measurement conditions. These results are mostly concentration- and viscosity-dependent.

### 2.10. Mechanical Properties

The sample size of 2.5×4×4 mm^3^ was cut down for the fracture strength measurement. The values of results can be uttered as the mean ± standard error. The force versus time graph is used to explain the hardness of the chitosan/TiO_2_ scaffold.

### 2.11. Density

Density measurement is important for physical property evaluation. Equation (1) was used to measure the density of scaffold that give the calculated ratio of the mass by volume of sample [21].

(1) $$\begin{array}{c} \rho =\frac{W}{\pi \times {\left(D/2\right)}^{2}\times H} \end{array}$$

where D is the diameter, *ρ* is density, H is the thickness, and W is the weight of the sample, respectively.

### 2.12. Swelling Behavior

PBS solution was used to check the swelling or water retention capability of the sample scaffold. The swelling capacity depends on the porosity of the sample and nature of materials. The swelling capacity of the sample was calculated by the following [21]:

(2) $$\begin{array}{c} Water\;\;Retention\left(\;\%\;\right)=\frac{W_{w}-W_{d}}{W_{d}}\times 100 \end{array}$$

where W_d_ is initial weight and W_w_ is the weight of the sample after swelling.

### 2.13. Biodegradation

The measurement of* in vitro *weight degradation of the chitosan/TiO_2_ scaffold was required to estimate the bioavailability of materials. The pieces of the sample were incubated in the PBS solution (pH 7.4) containing 1 × 10^4^ U/ml of lysozyme at room temperature for 14 days. After the interval of 7 and 14 days, degradation of the sample was recorded by using the following [21]:

(3) $$\begin{array}{c} Weight\;\;degraded\left(\;\%\;\right)=\frac{W_{f}-W_{0}}{W_{0}}\times 100 \end{array}$$

where* W*_*0*_ is initial weight and* W*_*f*_ is the final weight after degradation sample.

### 2.14. Cytotoxicity

To check the cytotoxicity and biocompatibility of chitosan/TiO_2_ scaffolds, fibroblast cell lines were maintained in the cell culture facility in MEM with 10% FBS and 100 U/ml penicillin–streptomycin. Before cell seeding, all the sample scaffolds were sterilized and placed in an incubator with cell culture for 2 hours with 5% CO_2_ and 85% humidity. The detached cells (1×10^5^ cells/100 *μ*l) were seeded dropwise on the surface of scaffolds for the investigation of cytocompatibility. The cell seeded scaffolds were placed in a humidified incubator at 37°C for 4 h for the cell attachment [22].

## 3. Results and Discussion

**Figure 2:**
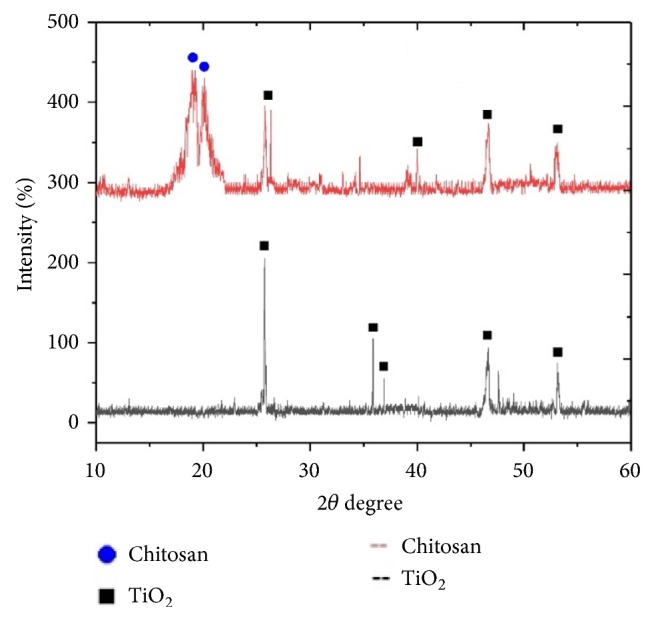
XRD analysis of nano-TiO_2_.

**Figure 3:**
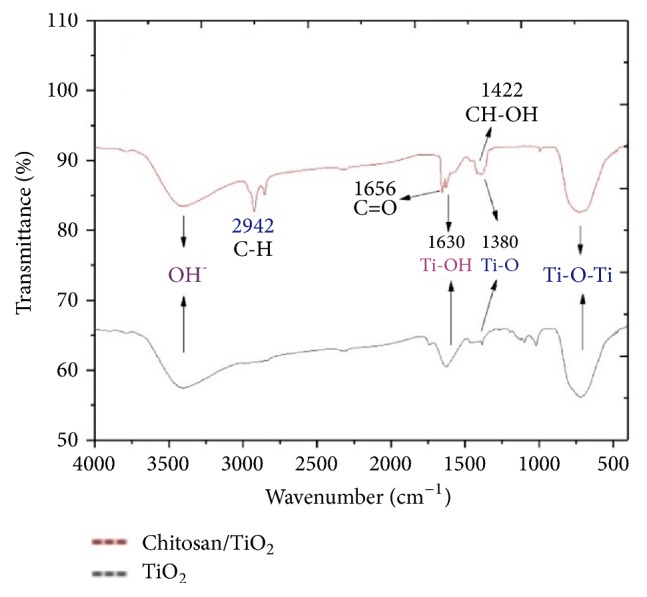
FTIR analysis of nano-TiO_2_.

The formation of nano-TiO_2_ was confirmed in XRD spectra (Figure 2). The particle size (4.48 nm) of TiO_2_ was determined by using Scherrer equation. The chitosan/TiO_2_ scaffold has shown a crystalline nature. The XRD of chitosan/TiO_2_ scaffold and TiO_2_ nanoparticles showed peaks at 25.7°, 35.8°, 36.9°, 40.2°, 46.6°, and 53.0° corresponding to TiO_2_ (crystalline) and broad phase from 18 to 21° corresponding to chitosan (slightly amorphous). The FTIR spectrum has confirmed the presence of relevant functional groups in the sample (Figure 3). There is a band present from 2800 to 3400 cm^−1^ in the sample due to OH stretching vibrations. The band present from 600 to 711 cm^−1^ represents Ti–O–Ti stretching bonding [23]. The peak present in the sample near 1630 cm^−1^ shows Ti-OH bending vibrations of adsorbed H_2_O molecules and peak at 1380cm^−1^ indicating Ti-O [24]. The presence of C-H, C=O, and CH-OH groups was confirmed at 2924, 1656, and 1422 cm^−1^, respectively [25].

**Figure 4:**
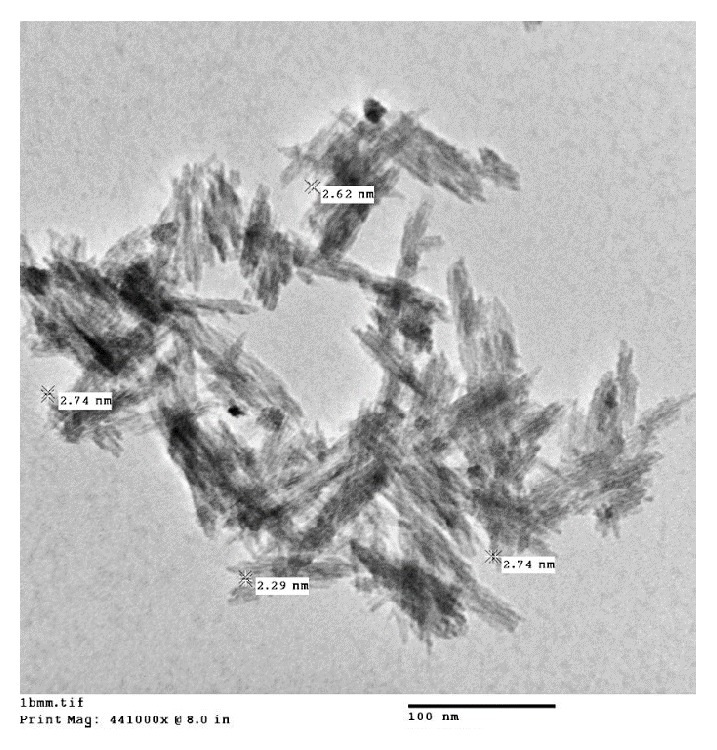
TEM image of TiO_2_ nanoparticles.

**Figure 5:**
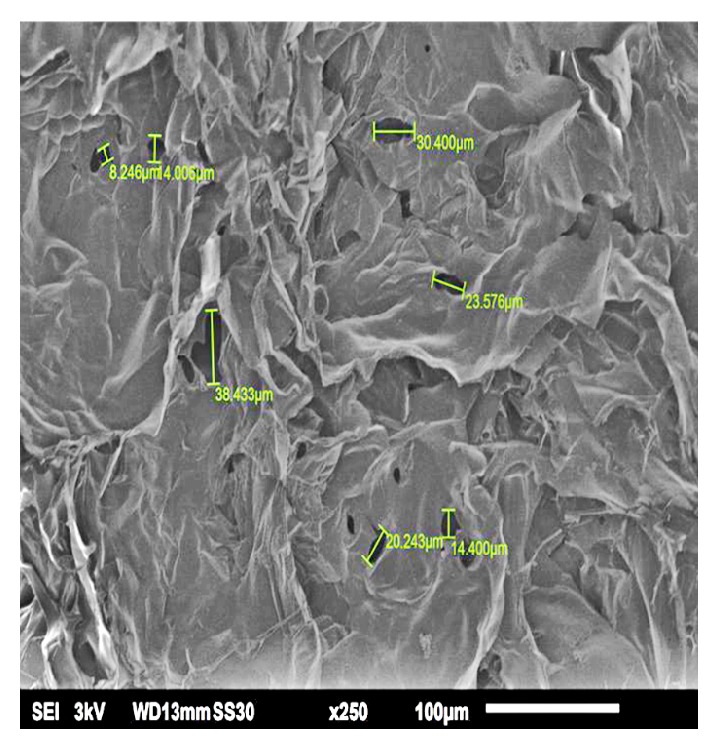
SEM images chitosan/TiO_2_ sample.

**Figure 6:**
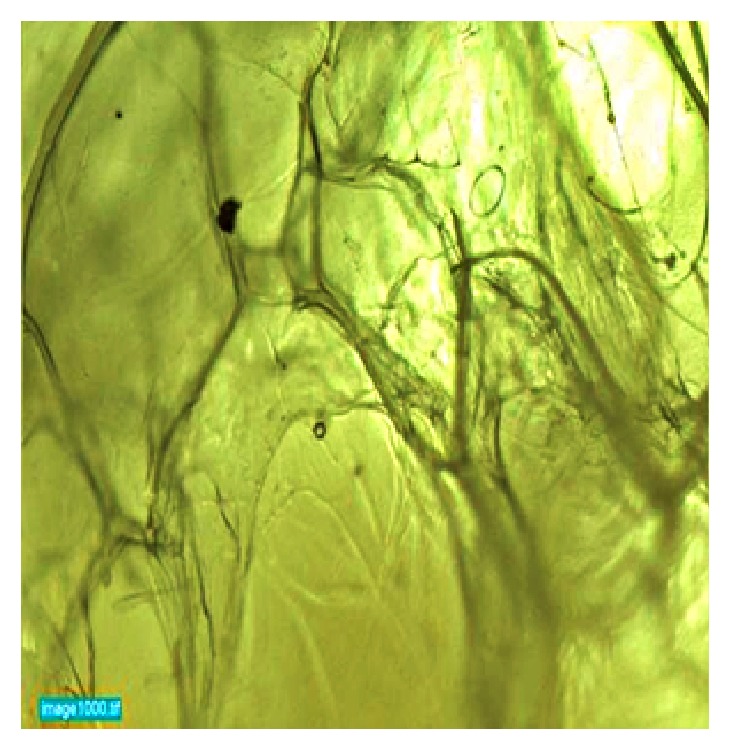
Microscopic image of chitosan/TiO_2_ sample.

**Figure 7:**
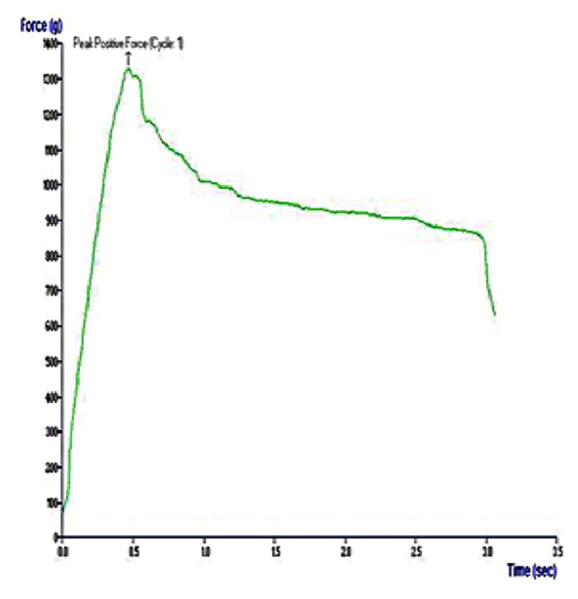
Force versus time graph for fracture strength.

**Figure 8:**
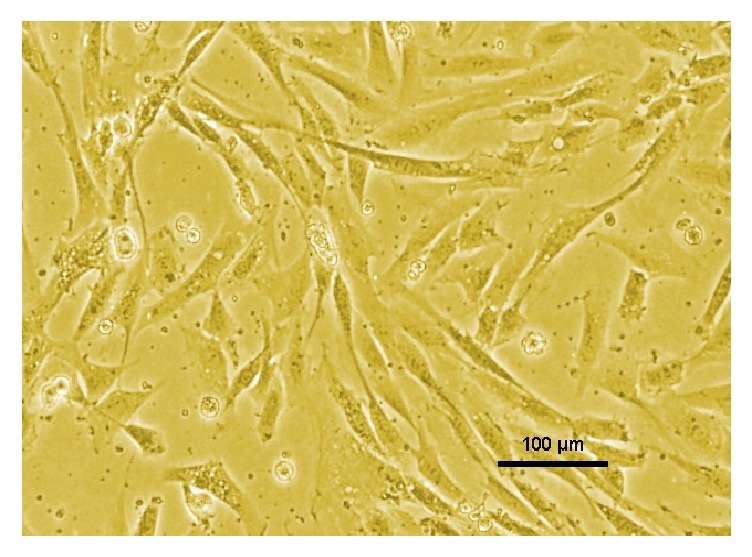
Fibroblast proliferation on chitosan/TiO_2_ scaffold.

**Figure 9:**
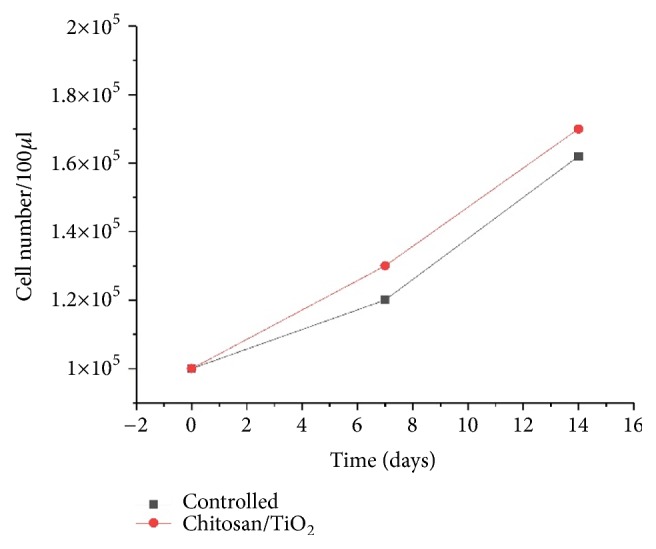
Cell proliferation on chitosan/TiO_2_ scaffolds as a function of time.

The hydrothermally synthesized nano-TiO_2_ is examined by TEM as shown in Figure 4. The TEM image of TiO_2_ is shown around 2 nm sized particles with irregular shape. TiO_2_ slurry does an interfacial interaction between chitosan and nanoparticles, which causes a nanoscale dispersion of TiO_2_ in the matrix. The interaction between TiO_2_ nanoparticles and chitosan depends on the charge state of the interface. Cationic chitosan is adsorbed on the surface of TiO_2_, driven by electrostatic interactions and steric effects; adsorption is strictly pH-dependent and creation of stable TiO_2_–chitosan composite [26]. Glass transition temperature (Tg) of chitosan (dry state) was found to be 118C confirmed by conventional DSC. The polymer-nanoparticles interactions play a key role in controlling the local dynamics of matrix and glass transition value of sample. The uniform dispersion of TiO_2_ in chitosan matrix improved the glass transition temperature (Tg) chitosan/TiO_2_ sample [27]. The well-interconnected, heterogeneous pore microstructures in sample scaffold are shown in Figure 5. Stretched pores were generated in the scaffold during lyophilization whose formation might be due to hydrogen bonding formation between polymer and nanoparticles and parallel ice crystal growth. SEM images of scaffold revealed mixed size of pores from 8.24 to 38.43*μ*m were found in the chitosan/TiO_2_ scaffold which are more relevant for tissue engineering because the pore sizes of bone, muscle, and skin vary from 20 to 300 *μ*m [20]. The pore size lower than 300*μ*m helps to proliferate osteoblast cell easily through the scaffold [28]. Porosity of scaffold examined by BET revealed a specific surface area (2.7752 m^2^g^−1^), pore specific surface area (3.8751 m^2^g^−1^), pore volume (0.0030 cm^3^g^−1^), and pore diameter 2.86 nm. Previously examined chitosan and chitosan-gelatin scaffolds [20] show less porosity than chitosan/TiO_2_ scaffold. The addition of TiO_2_ nanoparticles increased porosity of scaffold. The addition of nano-*f*-CNT in chitosan increased porosity [29], while the addition of nanosilica reduces the porosity [30]. The sample of chitosan/TiO_2_ as observed by fluorescence microscopy (Figure 6) revealed a low surface density of biomolecules and interconnected branched structure clearly shown in the image. The experimental density of chitosan/TiO_2_ scaffold found 1.2870g/cm^3^, which is more than chitosan and chitosan/gelatin scaffolds [20] and comparatively nearby the normal dry bone density (0.8 - 1.2 g/cm^3^). The density of TiO_2_ is 4.23 g/cm^3^ which is less than stainless steel 316L (7.9g/cm^3^), tantalum (16.6), and gold (19.3g/cm^3^); however, it is better than fat (0.94g/cm^3^), soft tissues (1.01-1.06g/cm^3^), glass (2.4-2.8g/cm^3^), bone (1-2 g /cm^3^), and aluminum oxide (3.98g/cm^3^). The density of chitosan is very low (0.15-0.30 g/cm^3^) so TiO_2_ is selected as a dopant to increase the density of scaffolds. The addition of nanobioglass and nanosilica enhanced the density of scaffolds [22, 30] but in this work we found that nano-TiO_2_ reduces the density of scaffold. The change in density of a scaffold depends upon the concentration of chitosan in a composition. The force-time graph shows the fracture strength with respect to breaking time of sample as shown in Figure 7. The doping of titanium oxide makes effective improvements in the strength of scaffold without producing toxicity, proved by International Agency for Research on Cancer (IARC). The force bearing capacity of the sample was 1347.5 N/m^2^ and breaking time was 0.58 sec that is better than chitosan scaffold, i.e., as previously described in [20], but less than CS/Alg, CS/Alg/nSiO_2_, chitosan/CNT, chitosan-PPy-Alg, and chitosan-gelatin scaffolds [20, 29–32]. Hence, the scaffold can be used for preparing implants of bioinert and lightweight, which makes brittle composite with low density. The physical and mechanical characteristics of scaffold depend on the viscosity, ingredient concentration, pH of matrix, temperature, and lyophilization. At room temperature, the observed water retention capacity of chitosan-TiO_2_ scaffold is found to be 24%, which is less than chitosan, chitosan-gelatin [20], chitosan-PPy-Alg [31], chitosan-alginate, chitosan-silica, [32], and chitosan-bioglass [22] scaffolds. The retention is also depending upon the porosity and nature of materials contained by the scaffolds. The percentage degradation of chitosan/TiO_2_ scaffold was very slow compared to other chitosan based scaffolds [20, 29–32], 12.24% after 7 days and 14.80% after 14 days. The degree of crystallinity is the major factor, which controls the hydrolysis rate. However, TiO_2_ supports apatite formation when it is encountered with Simulated Body Fluid [33]. TiO_2_ nanoparticles have shown similarity with nanostructured nature of microenvironment. They have ability to pass the biological barriers, enter into the cell nuclei, and affect the cell functions such as proliferation and differentiation. From results, we found that the incorporation of TiO_2_ in chitosan did not show any influence on fibroblast proliferation. Cytotoxicity and cell attachment studies showed the nanocomposites are nontoxic to an array of fibroblast cell line. The incorporation of TiO_2_ into biodegradable scaffolds may enhance cell seeding and hence the subsequent tissue growth [34]. After preselected time intervals (7 and 14 days), the number of cells increased as shown in Figures 8 and 9. Fibroblast cells were successfully grown on the surface of chitosan/TiO_2_ scaffolds.

## 4. Conclusion

The lyophilized sample of the scaffold was pale yellow in color, brittle, and inelastic. The scaffold fabricated using the freeze-drying technique exhibited high porosity and relatively low density with good mechanical properties. The remarkable adsorption or water retention capacity of TiO_2_ doped chitosan scaffold was noticed during investigation. The addition of TiO_2_ reduces the fast degradation of scaffold. The nanoparticles restricted the formation of the strong bond during sample preparation. Hence, it can be concluded that nanoparticles doped scaffolds can be used for preparing a biocompatible implant with lighter weight. The natural origin and biocompatible nature of chitosan support bone cells attachment, proliferation, and mineralization. Titanium oxide is inert in nature, so the combination of chitosan/TiO_2_ can be a good substitute for tissue engineering. Still a lot of research needs to be conducted for optimizing different parameters of scaffolds without compromising biodegradability and toxicity. Multidisciplinary approach for fabrication of new scaffolds with improved properties is highly desired.

## Data Availability

The data used to support the findings of this study are available from the corresponding author upon request.
